# Risk Factors in Patients with Diabetes Hospitalized for COVID-19: Findings from a Multicenter Retrospective Study

**DOI:** 10.1155/2021/3170190

**Published:** 2021-01-15

**Authors:** Yili Zhang, Juan Wang, Nannan Tan, KangJia Du, Kuo Gao, Jiacheng Zuo, Xiaoguang Lu, Yan Ma, Yong Hou, Quntang Li, Hongming Xu, Jin Huang, Qiuhua Huang, Hui Na, Jingwei Wang, Xiaoyan Wang, Yanhua Xiao, Junteng Zhu, Hong Chen, Zhang Liu, Mingxuan Wang, Linsong Zhang, Shuzhen Guo, Wei Wang

**Affiliations:** ^1^Beijing University of Chinese Medicine, Beijing, China; ^2^Institute of Basic Research in Clinical Medicine, China Academy of Chinese Medical Sciences, Beijing, China; ^3^The First Affiliated Hospital of Anhui University of Traditional Chinese Medicine, Hefei, Anhui, China; ^4^Chongqing Traditional Chinese Medicine Hospital, Chongqing, China; ^5^Department of Infectious Disease, Daqing Second Hospital, Daqing, Heilongjiang, China; ^6^Department of Traditional Chinese Medicine, The People's Hospital of GuangXi Zhuang Autonomous Region, Nanning, Guangxi, China; ^7^Department of Infectious Disease, Harbin Infectious Disease Hospital, Harbin, Heilongjiang, China; ^8^Department of Infectious Disease, Jinzhong Infectious Disease Hospital, Jinzhong, Shanxi, China; ^9^Department of Traditional Chinese Medicine, Mudanjiang Kangan Hospital, Mudanjiang, Heilongjiang, China; ^10^Department of Rehabilitation Medicine, The Affiliated Hospital of Putian College, Putian, Fujian, China; ^11^President's Office, The First Hospital of Qiqihar, Qiqihar, Heilongjiang, China; ^12^Department of Traditional Chinese Medicine, The First Hospital of Suihua City, Suihua, Heilongjiang, China; ^13^Department of Traditional Chinese Medicine, Suining Central Hospital, Suining, Sichuan, China; ^14^Department of Traditional Chinese Medicine, Hospital (T·C·M) Affiliated to Southwest Medical University, Luzhou, Sichuan, China

## Abstract

**Methods:**

In this multicenter retrospective study, patients with COVID-19 in China were included and classified into two groups according to whether they were complicated with diabetes or not. Demographic symptoms and laboratory data were extracted from medical records. Univariable and multivariable logistic regression methods were used to explore the risk factors.

**Results:**

538 COVID-19 patients were finally included in this study, of whom 492 were nondiabetes and 46 were diabetes. The median age was 47 years (IQR 35.0-56.0). And the elderly patients with diabetes were more likely to have dry cough, and the alanine aminotransferase, lactate dehydrogenase, Ca, and mean hemoglobin recovery rate were higher than the other groups. Furthermore, we also found the liver and kidney function of male patients was worse than that of female patients, while female cases should be paid more attention to the occurrence of bleeding and electrolyte disorders. Moreover, advance age, blood glucose, gender, prothrombin time, and total cholesterol could be considered as risk factors for COVID-19 patients with diabetes through the multivariable logistic regression model in our study.

**Conclusion:**

The potential risk factors found in our study showed a major piece of the complex puzzle linking diabetes and COVID-19 infection. Meanwhile, focusing on gender and age factors in COVID-19 patients with or without diabetes, specific clinical characteristics, and risk factors should be paid more attention by clinicians to figure out a targeted intervention to improve clinical efficacy worldwide.

## 1. Introduction

The morbidity of coronavirus infectious disease 2019 (COVID-19) caused by severe acute respiratory syndrome coronavirus 2 (SARS CoV-2) has been increasing, with more than 1 million confirmed cases and deaths worldwide [1]. Before June 27, 2020, a total of 9,808,340 cases of infection and 494,408 deaths have been reported worldwide [2]. Compared with severe acute respiratory syndrome (SARS) and Middle East respiratory syndrome (MERS), COVID-19 has a lower mortality among confirmed cases. However, patients with underlying comorbidities, including diabetes, hypertension, and coronary heart disease, are at greater risk of poor outcomes [3, 4].

Diabetic (DM) is one of the main causes of worldwide morbidity, which is expected to increase greatly in the next few decades [5]. The available evidence has shown that people with diabetes are more sensitive to certain infectious diseases, such as Staphylococcus aureus and Mycobacterium tuberculosis [6, 7], which may be caused by immune system disorders [8]. Furthermore, diabetes has been associated with a poor prognosis and increased pneumonia-associated mortality [9], and it was reported that DM patients with COVID-19 have higher nonsurvival rates (22% to 31%) than nondiabetic subgroups [10]. For individuals with COVID-19 and preexisting DM, a key challenge for clinicians is to improve outcomes in the face of uncertainty regarding the clinical characteristics and risk factors. However, a small retrospective single-center observational study conducted recently in China analyzed the clinical characteristics and outcomes of 48 patients with severe COVID-19 and diabetes [11] and found that there was no significant difference in the prevalence of complications between survivors of COVID-19 diabetes and nondiabetic patients. Thus, the specific risk factors and representative characteristics still need to be confirmed by larger sample size and well-designed clinical trials.

In this study, we performed a retrospective longitudinal, multi-centered study from a cohort of 538 confirmed COVID-19 cases enrolled from 11 hospitals in China. We aimed to describe the demographic features, clinical data, and outcomes of COVID-19 patients with DM. We also compared the characteristics and risk factors for the COVID diabetes patients with age and sex-matched patients without diabetes. We hope that these findings will provide new insights into risk stratification, disease management, and treatment strategies for patients with COVID-19 diabetes.

## 2. Methods

### 2.1. Study Design and Participants

This was a retrospective cohort study among patients with COVID-19 with diabetes. The patients with diabetes included in our study had a clear diagnosis of diabetes by their physicians based on electronic medical records. We included patients with the laboratory-confirmed SARS-CoV-2 infection at 11 hospitals in 6 provinces of China (appendix 1) from January 28 to February 25, 2020: the diagnosis of patients with COVID-19 according to the World Health Organization interim guidance [12]. The method of detection of SARS-CoV-2 using throat swabs and RT-PCR was reported previously [13]. Diabetes was defined according to the guidelines of American Diabetes Association [14]. The outcomes were the clinical characteristics, laboratory findings, and incidence of complications in COVID-19 patients with and without diabetes.

### 2.2. Ethics Statement

National Administration of Traditional Chinese Medicine, Administration of Traditional Chinese Medicine of the above provinces, and the institutional board of 11 participating setting approved this study. Due to the urgent need to collect data on this emerging infectious disease, the requirement for written informed consent was waived.

### 2.3. Data Collection and Statistical Analysis

Demographic data, exposure history, symptoms and signs, laboratory findings, and chest CT scan data were extracted. The date of disease onset was defined as the day when the first symptom showed up. All data were reviewed by the research team and checked by two physicians and a third researcher.

The classification variable was expressed as frequency and percentage (%), and if the continuous variable was not a normal distribution, the median (IQR) was used. The *χ*^2^ test or Fisher exact test was used to compare the classified variables between the two groups, and Student's *t*-test or Mann–Whitney *U* test was used as appropriate to analyze continuous variables. In addition, we conducted an in-depth analysis of the age and gender dimensions of the diabetes and nondiabetes groups. To explore the risk factors associated with COVID-19 diabetes, univariate and multivariate logistic regression models were used. All statistical analysis was carried out with SPSS 21.0 software. Bidirectional alpha less than 0.05 was considered statistically significant.

## 3. Results

Among the 538 hospitalized patients with COVID-19 diabetes in six provinces of China (supplementary Tables 1), the median age was 47 years (IQR 35.0-56.0), and there was no significant difference in the ratio of male to female (supplementary Tables 3). It was reported that some patients suffered from complications, of which hypertension was the most common complication, followed by coronary heart disease and cerebrovascular disease (supplementary Tables 2 and supplementary Tables 3). The most common symptoms on admission were fever and cough, followed by dry cough and fatigue (supplementary Tables 3). Major laboratory markers were tracked from the onset of the disease (supplementary Tables 3).

There were significant differences in age and sex between diabetic and nondiabetic patients. The median age of the diabetic group was older than that of the nondiabetic group (Table 1). Among the laboratory main indicators, diabetic patients had higher baseline total cholesterol, triglyceride, cystatin C, and anion gap (Table 1).

In addition, we compared the baseline between male and female patients in each group. In the diabetic group, the levels of lipase, potassium, and activated partial thromboplastin time in females were higher than those in males, while the width of the platelet distribution in females was smaller than that in males. However, the liver and kidney function of male patients was worse than that of female patients in terms of *γ*-glutamyl transpeptidase, creatinine, and urea (Table 2). In the nondiabetes group, women were more likely to show signs of fatigue and anorexia and had lower hemoglobin, average hemoglobin, and average hemoglobin than men. The liver and kidney function of male patients also seemed to be poor, with high levels of *γ*-glutamyl transpeptidase, uric acid, creatinine, cystatin C, and low glomerular filtration rate (Table 2). We also compared the clinical features in the perspective of different ages, and the results showed that elderly patients with diabetes were more likely to have dry cough, and the alanine aminotransferase, lactate dehydrogenase, Ca, and mean hemoglobin recovery rate were higher than the other groups (Table 3).

For the risk factor analysis, age, sex, triglyceride, prothrombin time, cystatin C, uric acid, *α*-hydroxybutyrate dehydrogenase, anion gap, total cholesterol, and glucose were associated with DM cases based on the univariable analysis (Table 4). In the multivariable logistic regression analysis, we found that glucose, advance age, gender, prothrombin time, and total cholesterol were associated with diabetes (Table 4). When adjusting for the study center, our generalized linear model also showed similar results (supplementary Tables 4).

## 4. Discussion

It was generally believed that DM was associated with a general increase in mortality and morbidity of infectious diseases, although the epidemiological data showed a surprising scarcity. However, it seems certain that DM can easily lead to certain types of infection and death [5].

In this study, we found that age, gender, and laboratory indicators (total cholesterol, triglyceride, cystatin C, sodium ion, and anion gap) were markedly correlated with the DM. Furthermore, the indicators of the liver and kidney function, inflammation, and immune system in patients with DM were analyzed from the perspective of gender, suggesting that the health surveillance and prevention of male patients with DM should be strengthened to avoid the deterioration of the liver and kidney function, while female cases should be paid more attention to the occurrence of bleeding and electrolyte disorders.

In view of the importance of the above factors, advance age, blood glucose, gender, prothrombin time (PT), and total cholesterol could be considered as risk factors for COVID-19 patients with DM through the multivariable logistic regression model, which was helpful to the prognosis of COVID-19 patients complicated with DM.

Angiotensin-converting enzyme-2 (ACE-2) seemed to be one of the most important factors in the study of cellular and molecular mechanisms that may increase the risk of COVID-19 in patients with diabetes [15]. ACE-2 has been determined from human heart failure and lymphoma cDNA libraries [16] and was proved to be the receptor of SARS-CoV-2 [17]. ACE-2 has also been served as the cellular entry point for the virus SARS-CoV-2 [18]. The affinity of SARS-CoV-2 to ACE-2 was even higher than that of SARS-CoV-2 [19]. Recent publications have shown that old age was an independent predictor of mortality in patients with COVID-19 [4]. It has previously been reported the age-dependent decrease of the cellular and humoral immune function in elderly patients, especially in terms of the adaptive immune function [20]. In our study, people with diabetes were older than those without diabetes. In addition, the high risk of elderly patients with diabetes may be attributed to their overall poor health and an increase in the number of complications.

In addition, our study found that there were gender differences in patients with COVID complicated with diabetes. ACE-2 was located on the X chromosome, while there was only one allele in men and two alleles in women [21]. Furthermore, estrogen was believed to upregulate the expression of ACE-2 [22], and it provided premenopausal women with the advantage of two alleles and upregulation of estrogen, making ACE-2 deficiency and its rebound less likely in the event of a viral attack. Therefore, the possibility of male infection was not significantly reduced, but the pathophysiological manifestation of the disease was more serious. Marked gender differences in the disease have been confirmed in other large observational studies reporting 37% women in 6,232 patients with COVID, and a previous study reported 40% female patients in 8,910 COVID-19 patients requiring hospital admission and furthermore reported improved survival in female patients, independent of older age [23].

Recent studies have reported that the level of blood glucose control directly affects the immune response and status of the human body [24], which was in line with our study. Diabetic patients have low immunity and are prone to increase the risk of disease. Once infected, it is likely to aggravate the condition of diabetic patients, further increase the difficulty of blood glucose control, and more easily aggravate the infection, thus leading to cytokine storm and acute inflammatory response. Inflammation is closely related to the occurrence and development of diabetes [25, 26]. A clinical study found that COVID-19 patients without other comorbidities but diabetes that had higher serum levels of inflammation-related biomarkers such as IL-6 were susceptible to a cytokine storm, leading to rapid deterioration of COVID-19 [27]. Inflammatory cytokines can cause structural and functional abnormalities of endothelial cells, leading to insulin transport disorders in human tissues and cells and thus lead to insulin resistance. Meanwhile, inflammatory cytokines may lead to structural changes and dysfunction of *β* cell, promote apoptosis of *β* cell, cause insufficiency of insulin secretion, and eventually lead to the rise of blood glucose. In the later stage of life for COVID-19 with diabetes, “cytokine storm” will start rapidly and enter the state of multiple organ failure, including liver and kidney failure [28]. When the liver tissue of patients with COVID-19 is extensively damaged, the ability of liver cells to use glucose to synthesize glycogen is decreased, which leads to the aggravation of insulin resistance and the increase of blood glucose.

Considering the hemostatic parameters analyzed in our study, prothrombin time was significantly increased in both T2DM patients and control groups, indicating that T2DM was in a hypercoagulable state which was mainly affected by high average glucose levels. The results were consistent with previous studies, indicating that the synthesis of TF and FVII is more prominent in diabetes [29, 30]. Current evidence demonstrated that hyperglycemia can lead to hyperfibrinogenemia in patients with diabetes and activate the coagulation cascade and increase the formation of thrombin and fibrinogen degradation products, which may stimulate the synthesis of fibrinogen in the liver. The level of fibrinogen and the factors in the internal pathway were increased in patients with diabetes, which were the determinants of APTT. PT tests are standard screening tests for the function of the coagulation system, and their utility in monitoring therapeutic anticoagulation is widely accepted. As in T2DM, people with metabolic syndrome display a clotting factor pattern that promotes thrombosis or prevents thrombolysis [31].

Emerging evidence suggests that dysregulation of lipid transport may contribute to some of these complications [32]. Specifically, it was hypothesized that changes in the amount and composition of high-density lipoprotein (HDL) that occurs with COVID-19 can significantly decrease the anti-inflammatory and antioxidative function of HDL and could contribute to pulmonary inflammation [33]. In the present study, cholesterol, an insoluble eukaryotic lipid found throughout the body's membrane, was another risk factor worthy of attention. Lung cells in the lung load and unload cholesterol together with macrophages are associated with lung disease [34] and lung function [35]. Aspect of the SARS-CoV-1 entry, a closely related virus, suggests a potential mechanism for cholesterols effects. In cultured cells, SARS-CoV-1 is thought to utilize both a cholesterol-dependent endocytic pathway through monosialotetrahexosylganglioside1 (GM1) lipid domains and a cell surface mechanism [36]. Cholesterol is needed to form GM1 lipid rafts used in the endocytic process. Furthermore, the cholesterol localizes ACE2 to detergent-resistant membranes (DRMs) [37], which are similar in composition to GM1 lipid domains but not the same. Hence, researchers hypothesized that the SARS-CoV-2 entry depends on cholesterol loading into the lung tissue.

Despite the importance of the aforementioned findings, the current study, however, has some limitations. Firstly, data collection relied on electronic medical records. Some important indicators were not tested in all patients. Thus, the missing data might lead to bias. Secondly, the relatively small sample size might influence the interpretation of our findings. Finally, the present report focuses only on short-term prognosis (i.e., 7 days after admission), and one cannot exclude the possibility that diabetes characteristics prior to admission could be associated with severe COVID-19 outcomes in the longer term.

Collectively, our findings suggest there is work for all of us. As an observational study covering 11 hospitals in 6 provinces across the country, it has undoubtedly provided a major piece of the complex puzzle linking diabetes and COVID-19 infection: people with diabetes should be more attentive than others to take self-protective actions, particularly paying more attention to the levels of glucose, PT, and total cholesterol. We believe that it will contribute to the prognosis and symptomatic treatment of COVID-19 patients with DM.

## Figures and Tables

**Table 1:** Demographic, clinical, laboratory, and radiographic findings of COVID-19 patients on admission.

	Total	Patients without diabetes	Patients with diabetes	*p* value
	(*n* = 538)	(*n* = 492)	(*n* = 46)	
Demographics				
Age, years	47.00 (35.00-56.00)	45.00 (33.00-56.00)	55.50 (49.00-62.25)	<0.0001
Sex				
Female	260 (48.33%)	246 (50%)	14 (30.43%)	0.011
Male	278 (51.67%)	246 (50%)	32 (69.57%)	
Laboratory findings				
Direct bilirubin, umol/L				
<1	25 (4.65%)	25 (5.08%)	0 (0%)	0.001
1-14	422 (78.44%)	384 (78.05%)	38 (82.61%)	
>14	6 (1.12%)	3 (0.61%)	3 (6.52%)	
Total cholesterol, mmol/L	4.10 (3.54-4.81)	4.05 (3.53-4.77)	4.51 (4.08-5.32)	0.019
Triglyceride, mmol/L	1.38 (0.95-2.05)	1.35 (0.91-2.01)	1.85 (1.40-2.64)	0.002
<0.24	0 (0%)	0 (0%)	0 (0%)	0.054
0.24-1.86	219 (40.71%)	206 (41.87%)	13 (28.26%)	
>1.86	98 (18.22%)	86 (17.48%)	12 (26.09%)	
Creatinine, umol/L				
<53	124 (23.05%)	113 (22.97%)	11 (23.91%)	<0.0001
53-97	313 (58.18%)	289 (58.74%)	24 (52.17%)	
>97	16 (2.97%)	10 (2.03%)	6 (13.04%)	
Cystatin C, mg/L	0.92 (0.80-1.11)	0.91 (0.78-1.10)	1.00 (0.89-1.15)	0.041
Na, mmol/L	139.00 (137.00-141.00)	139.00 (137.00-141.00)	139.60 (138.00-141.50)	0.041
Cl, mmol/L				
<99	62 (11.52%)	51 (10.37%)	11 (23.91%)	0.045
99-110	373 (69.33%)	343 (69.72%)	30 (65.22%)	
>110	3 (0.56%)	3 (0.61%)	0 (0%)	
Anion gap, mmol/L	10.50 (8.85-12.20)	10.20 (8.50-12.00)	13.00 (9.40-16.30)	0.011
<10	45 (8.36%)	42 (8.54%)	3 (6.52%)	0.011
10-14	57 (10.59%)	52 (10.57%)	5 (10.87%)	
>14	7 (1.3%)	4 (0.81%)	3 (6.52%)	

Data are median (IQR), *n* (%), or *n*/*N* (%). *p* values were calculated by the Mann–Whitney *U* test, *χ*^2^ test, or Fisher's exact test, as appropriate.

**Table 2:** Clinical characteristics and laboratory findings of COVID-19 patients with and without diabetes in different gender.

Patients with diabetes				
	Total (*n* = 45)	Male (*n* = 32)	Female (*n* = 14)	*p* value
Laboratory findings				
*γ*-Glutamyl transpeptidase, IU/L	38.55 (21.00-70.25)	56.40 (24.00-72.00)	25.00 (14.00-38.25)	0.028
Lipase, U/L	44.30 (38.60-56.70)	40.70 (35.35-44.65)	53.35 (44.48-80.80)	0.053
Urea, mmol/L	4.10 (2.94-5.72)	5.10 (3.12-6.34)	2.95 (2.57-4.30)	0.015
Creatinine, umol/L	63.00 (50.15-84.40)	71.10 (57.20-89.40)	55.11 (41.38-70.50)	0.034
K, mmol/L	3.94 (3.62-4.12)	3.88 (3.50-4.05)	4.08 (3.79-4.31)	0.035
Platelet distribution width	14.80 (11.85-16.10)	15.90 (12.30-16.20)	12.00 (10.25-15.10)	0.051
Activated partial thromboplastin time, s	29.40 (25.75-34.60)	26.60 (23.83-30.86)	33.40 (30.48-35.50)	0.054
Patients without diabetes				
	Total (*n* = 492)	Male (*n* = 246)	Female (*n* = 246)	*p* value
Symptoms and signs				
Fatigue	92 (18.70%)	35 (14.23%)	57 (23.17%)	0.011
Anorexia	67 (13.62)	23 (9.35%)	44 (17.88%)	0.006
Laboratory findings				
*γ*-Glutamyl transpeptidase, IU/L	30.00 (19.00-56.00)	32.85 (20.85-56.00)	25.45 (17.00-56.43)	0.017
Uric acid, umol/L	276.36 (216.00-348.04)	296.60 (228.23-369.00)	265.30 (204.04-329.20)	0.01
Creatinine, umol/L	61.61 (52.00-75.00)	65.00 (55.00-76.76)	59.70 (49.26-71.00)	0
Cystatin C, mg/L	0.91 (0.78-1.10)	0.99 (0.80-1.19)	0.88 (0.75-1.01)	0.007
Glomerular filtration rate	111.28 (103.67-120.58)	108.74 (100.45-114.60)	116.4 (104.80-127.64)	0.015
Hemoglobin, g/L	134.00 (121.00-148.00)	137.00 (123.00-148.00)	130.00 (119.50-147.50)	0.038
Mean hemoglobin, pg	30.30 (29.18-31.40)	30.50 (29.40-31.50)	30.10 (28.90-31.30)	0.038
Mean hemoglobin concentration, g/L	335.00 (326.00-344.00)	337.00 (326.25-345.00)	334.00 (325.00-342.00)	0.009

**Table 3:** Clinical characteristics and laboratory findings of COVID-19 patients with and without diabetes in different age groups.

Patients with diabetes				
	Total (*n* = 46)	Elderly (≥60 yr) (*n* = 31)	Non-elderly (<60 yr) (*n* = 15)	*p* value
Symptoms and signs				
Dry cough	9 (19.57%)	9 (29.03%)	0	0.02
Laboratory findings				
Alanine aminotransferase, IU/L	24.00 (17.50-17.50)	28.00 (28.00-28.00)	18.06 (13.75-26.50)	0.033
Alanine aminotransferase, IU/L	23.00 (17.50-17.50)	26.00 (26.00-26.00)	18.00 (15.25-24.00)	0.019
Lactate dehydrogenase, IU/L	183.50 (150.53-150.53)	191.50 (191.50-191.50)	164.00 (138.23-185.00)	0.031
Ca, mmol/L	2.29 (2.12-2.12)	2.34 (2.34-2.34)	2.19 (2.07-2.30)	0.035
Mean hemoglobin, pg	30.60 (29.20-29.20)	30.75 (30.75-30.75)	29.60 (28.60-31.30)	0.051
Patients without diabetes				
	Total (*n* = 492)	Elderly (≥60 yr) (*n* = 407)	Non-elderly (<60 yr) (*n* = 85)	*p* value
Clinical characteristics				
Nonsevere	447 (90.85%)	382 (93.86%)	65 (76.47%)	<0.0001
Severe	45 (9.15%)	25 (6.14%)	20 (23.53%)	
Comorbidity				
Hypertension	69 (14.02%)	65 (15.97%)	4 (4.71%)	0.007
Symptoms and signs				
Fever	197 (40.04%)	151 (37.1%)	46 (54.12%)	0.004
Cough	251 (51.02%)	196 (48.16%)	55 (64.71%)	0.006
Stuffiness	19 (3.86%)	19 (4.67%)	0 (0%)	0.042
Asthma	58 (11.79%)	40 (9.83%)	18 (21.18%)	0.003
Laboratory findings				
Albumin, g/L	39.60 (35.35-43.90)	39.77 (35.90-43.90)	38.30 (34.25-43.97)	0.049
Ischemia modified albumin, U/ml	75.67 (69.33-80.75)	78.88 (69.99-81.83)	68.48 (66.91-69.94)	0.012
Low-density lipoprotein, mmol/L	2.41 (1.94-3.00)	2.42 (1.94-2.96)	2.14 (1.94-3.24)	0.044
Complement C1q, mg/L	164.41 (138.90-166.53)	164.41 (141.39-169.65)	150.14 (134.51-.)	0.03
*α*-L-fucosidase, U/L	25.00 (21.00-29.80)	25.00 (21.00-29.80)	25.10 (21.25-29.50)	0.01
Ca, mmol/L	2.23 (2.11-2.35)	2.24 (2.13-2.35)	2.14 (2.08-2.28)	0.043
Neutrophil percentage,%	65.40 (56.73-73.98)	65.30 (56.60-73.45)	68.30 (61.20-81.70)	0.014
Monocyte percentage, %	7.50 (5.90-9.40)	7.55 (6.10-9.50)	6.30 (4.70-8.80)	0.005
Hemoglobin, g/L	134.00 (121.00-148.00)	134.00 (121.00-148.00)	134.00 (121.00-152.00)	0.015
D-dimer, ug/mL	0.40 (0.24-0.80)	0.41 (0.24-0.79)	0.36 (0.21-1.25)	0.033
C-reactive protein, mg/L	7.70 (2.26-25.98)	7.30 (2.24-23.63)	11.55 (4.26-61.92)	0.045

**Table 4:** Risk factors associated with COVID-19 patients with diabetes.

	Univariable OR (95% CI)	*p* value	Multivariable OR (95% CI)	*p* value
Demographics and clinical characteristics				
Age	1.05 (1.03, 1.07)	<.0001	1.04 (1.00, 1.13)	0.4201
Sex	0.50 (0.27, 0.94)	0.0315	4.04 (0.34, 48.40)	0.271
Laboratory findings				
Triglyceride, mmol/L				
<0.24				
0.24-1.86	9.84 (1.89, 51.34)	0.0067		
>1.86	0.98 (0.64, 1.49)	0.9187		
Prothrombin time, s				
<9.5				
9.5-14.5	0.72 (0.53, 0.98)	0.0339	0.60 (0.27, 1.33)	0.2084
>14.5	0.68 (0.17 2.84)	0.5997		
Cystatin C, mg/L	4.01 (1.17, 13.71)	0.0271		
Uric acid, umol/L				
<140	0.98 (0.95, 1.02)	0.3509		
140-440	1.00 (1.00, 1.01)	0.7094		
>440	1.02 (1.00, 1.03)	0.0473		
*α*-Hydroxybutyrate dehydrogenase, IU/L				
<72				
72-182	0.98 (0.95, 1.00)	0.0797		
>182	1.01 (1.00, 1.02)	0.0419		
Anion gap, mmol/L	1.47 (1.14, 1.90)	0.0033		
Total cholesterol, mmol/L				
<2.8	0.81 (0.03, 24.33)	0.9037		
2.8-6	1.83 (1.10, 3.05)	0.0077	1.31 (0.35, 4.95)	0.69
>6	0.95 (0.62, 1.45)	0.8054		
Glucose, mmol/L	7.33 (1.66, 32.31)	0.0085	14.85 (0.74, 299.81)	0.0785

OR: odds ratio.

## Data Availability

All data used to support the findings of this study are included within the article.

## Supplementary Materials

Supplementary 1 Supplementary Table 1: distribution of source hospitals for enrolled patients. Supplementary Table 2: comorbidities of enrolled patients. Supplementary Table 3: demographic, clinical, laboratory, and radiographic findings of COVID-19 patients on admission. Supplementary Table 4: risk factors associated with COVID-19 patients with diabetes in the generalized linear model.
