# Supplementary material for: Risk Factors in Patients with Diabetes Hospitalized for COVID-19: Findings from a Multicenter Retrospective Study
Source: J Diabetes Res. 2021 Jan 15;2021:3170190. doi: 10.1155/2021/3170190 (PMC7847355; doi:10.1155/2021/3170190)
Supplement: Supplementary 1 — Supplementary Table 1: distribution of source hospitals for enrolled patients. Supplementary Table 2: comorbidities of enrolled patients. Supplementary Table 3: demographic, clinical, laboratory, and radiographic findings of COVID-19 patients on admission. Supplementary Table 4: risk factors associated with COVID-19 patients with diabetes in the generalized linear model. [file 3170190.f1.docx]

| **Supplementary Table 1: Distribution of source hospitals for enrolled patients** | | |
| --- | --- | --- |
| **Hospital** | **Case** | **Percentage** |
| Suining Central Hospital in Sichuan Province Suining city | 10 | 1.86% |
| Kang'an Hospital in Mudanjiang City Heilongjiang Province | 18 | 3.35% |
| The 2th Hospital in Daqing City Heilongjiang | 19 | 3.53% |
| The First Hospital of Qiqihar in Heilongjiang Province | 28 | 5.20% |
| The Infectious Disease Hospital of Jinzhong City in Shanxi Province | 32 | 5.95% |
| The people's hospital of guangzi zhuang autonomous region | 37 | 6.88% |
| No.1 Hospital In Suihua City in Heilongjiang Province | 45 | 8.36% |
| Anhui Traditional Chinese Medicine Hospital in Heifei city Anhui Province | 47 | 8.73% |
| The Affiliated Hospital(group) of Putian University in Putian city Fujian Province | 51 | 9.48% |
| The Infectious Hospital of Harbin in Heilognjiang Province | 68 | 12.64% |
| The Hospital Affiliated of Southwest Medical University in Luzhou city Sichuan Province | 183 | 34.02% |
| Total | 538 | 100.00% |

| **Supplementary Table 2: Comorbidities of enrolled patients** | |
| --- | --- |
| **Variable** | **Count** |
| Hypertension | 78 |
| Coronary heart disease | 15 |
| Cerebral vascular disease | 9 |
| Lung diseases | 5 |
| Other | 64 |
| Hypertension + CHD | 7 |
| Hypertension + other | 10 |
| Hypertension + Cerebral vascular disease | 4 |
| Hypertension + CHD + other | 2 |

Abbreviation: CHD, coronary heart disease.

| **Supplementary Table 3: Demographic, clinical, laboratory, and radiographic findings of COVID-19 patients on admission** | | | | |
| --- | --- | --- | --- | --- |
|  | **Total** | **Patients without diabetes** | **Patients with diabetes** | **p value** |
|  | **(n=538)** | **(n=492)** | **(n=46)** |  |
| **Demographics** | | | | |
| Age, years | 47.00(35.00-56.00) | 45.00(33.00-56.00) | 55.50(49.00-62.25) | <0.0001 |
| Sex |  |  |  |  |
| Female | 260(48.33%) | 246(50.00%) | 14(30.43%) | 0.011 |
| Male | 278(51.67%) | 246(50.00%) | 32(69.57%) |  |
| Non-severe | 485(90.15%) | 447(90.85%) | 38(82.61%) | 0.073 |
| Severe | 53(9.85%) | 45(9.15%) | 8(17.39%) |  |
| Epidemic area exposure history | 162(30.11%) | 150(30.49%) | 12(26.09%) | 0.534 |
| History of close contact with patients | 355(65.99%) | 322(65.45%) | 33(71.74%) | 0.399 |
| **Comorbidities** | | | | |
| Hypertension | 78(14.50%) | 69(14.02%) | 9(19.57%) | 0.307 |
| Coronary heart disease | 15(2.79%) | 14(2.85%) | 1(2.17%) | 0.791 |
| Cerebral vascular disease | 9(1.67%) | 8(1.63%) | 1(2.17%) | 0.782 |
| Lung diseases | 5(0.93%) | 5(1.02%) | 0(0%) | 0.492 |
| Others (thyroid disease, enteritis, fracture, etc.) | 64(11.90%) | 61(12.40%) | 3(6.52%) | 0.239 |
| **Symptoms and signs** | | | | |
| Fever | 218(40.52%) | 197(40.04%) | 21(45.65%) | 0.458 |
| Fatigue | 102(18.96%) | 92(18.70%) | 10(21.74%) | 0.615 |
| Cough | 271(50.37%) | 251(51.02%) | 20(43.48%) | 0.328 |
| Dry cough | 167(31.04%) | 158(32.11%) | 9(19.57%) | 0.079 |
| Stuffiness | 21(3.90%) | 19(3.86%) | 2(4.35%) | 0.871 |
| Runny nose | 18(3.35%) | 16(3.25%) | 2(4.35%) | 0.693 |
| Anorexia | 74(13.75%) | 67(13.62%) | 7(15.22%) | 0.763 |
| Sore throat | 34(6.32%) | 31(6.30%) | 3(6.52%) | 0.953 |
| Diarrhea | 53(9.85%) | 50(10.16%) | 3(6.52%) | 0.428 |
| Asthma | 64(11.90%) | 58(11.79%) | 6(13.04%) | 0.802 |
| Chills | 15(2.79%) | 12(2.44%) | 3(6.52%) | 0.108 |
| **Laboratory findings** | | | | |
| Alanine aminotransferase,IU/L | 24.00(16.00-38.50) | 24.00(16.00-38.00) | 24.00(17.50-51.50) | 0.412 |
| ＜7 | 6(1.12%) | 5(1.02%) | 1(2.17%) | 0.3 |
| 7-40 | 319(59.29%) | 295(59.96%) | 24(52.17%) |  |
| ＞40 | 100(18.59%) | 88(17.89%) | 12(26.09%) |  |
| Aspartate aminotransferase,IU/L | 21.95(17.00-31.00) | 21.00(17.00-31.00) | 23.00(17.50-30.50) | 0.462 |
| ＜12 | 14(2.6%) | 14(2.85%) | 0(0%) | 0.49 |
| 12-35 | 350(65.06%) | 318(64.63%) | 32(69.57%) |  |
| ＞35 | 60(11.15%) | 55(11.18%) | 5(10.87%) |  |
| γ-glutamyl transpeptidase,IU/L | 30.00(19.28-57.44) | 30.00(19.00-56.00) | 38.55(21.00-70.25) | 0.157 |
| ＜7 | 1(0.19%) | 1(0.2%) | 0(0%) | 0.318 |
| 7-45 | 276(51.3%) | 254(51.63%) | 22(47.83%) |  |
| ＞45 | 145(26.95%) | 127(25.81%) | 18(39.13%) |  |
| Alkaline phosphatase,IU/L | 67.00(51.00-86.80) | 66.45(51.00-86.70) | 75.30(51.00-89.00) | 0.456 |
| ＜50 | 93(17.29%) | 84(17.07%) | 9(19.57%) | 0.141 |
| 50-75 | 166(30.86%) | 156(31.71%) | 10(21.74%) |  |
| ＞75 | 162(30.11%) | 142(28.86%) | 20(43.48%) |  |
| Cholinesterase,IU/L | 7445.00(6205.00-8540.00) | 7402.00(6193.25-8556.75) | 7651.00(6518.00-8304.00) | 0.76 |
| ＜4000 | 8(1.49%) | 7(1.42%) | 1(2.17%) | 0.934 |
| 4000-12000 | 206(38.29%) | 182(36.99%) | 24(52.17%) |  |
| ＞12000 | 1(0.19%) | 1(0.2%) | 0(0%) |  |
| Lactate dehydrogenase,IU/L | 192.00(154.50-231.57) | 193.00(155.00-232.00) | 183.50(150.53-225.75) | 0.651 |
| 20-240 | 293(54.46%) | 264(53.66%) | 29(63.04%) | 0.315 |
| ＞240 | 80(14.87%) | 75(15.24%) | 5(10.87%) |  |
| α-hydroxybutyrate dehydrogenase,IU/L | 157.77(133.75-200.65) | 159.77(134.14-200.09) | 145.50(123.75-245.50) | 0.602 |
| ＜72 | 3(0.56%) | 3(0.61%) | 0(0%) | 0.83 |
| 72-182 | 108(20.07%) | 96(19.51%) | 12(26.09%) |  |
| ＞182 | 55(10.22%) | 49(9.96%) | 6(13.04%) |  |
| ApoA-1,g/L | 0.95(0.75-1.11) | 0.95(0.75-1.16) | 0.93(0.73-1.04) | 0.567 |
| ＜1 | 68(12.64%) | 59(11.99%) | 9(19.57%) | 0.842 |
| 1-1.6 | 49(9.11%) | 42(8.54%) | 7(15.22%) |  |
| ＞1.6g/L | 2(0.37%) | 2(0.41%) | 0(0%) |  |
| Apo-B,g/L | 0.82(0.61-1.01) | 0.82(0.61-1.01) | 0.87(0.58-1.02) | 0.912 |
| ＜0.6g/L | 26(4.83%) | 22(4.47%) | 4(8.7%) | 0.92 |
| 0.6-1.1g/L | 74(13.75%) | 64(13.01%) | 10(21.74%) |  |
| ＞1.1g/L | 18(3.35%) | 16(3.25%) | 2(4.35%) |  |
| Total bile acid,umol/L | 3.80(2.52-6.28) | 3.80(2.53-6.30) | 3.40(2.51-5.40) | 0.638 |
| ＜20 | 301(55.95%) | 269(54.67%) | 32(69.57%) | 0.55 |
| ＞20 | 3(0.56%) | 3(0.61%) | 0(0%) |  |
| Total protein,g/L | 67.20(62.00-73.68) | 67.80(61.81-73.74) | 66.00(62.62-71.87) | 0.676 |
| ＜65 | 180(33.46%) | 163(33.13%) | 17(36.96%) | 0.47 |
| 65-83 | 278(51.67%) | 255(51.83%) | 23(50%) |  |
| ＞83 | 18(3.35%) | 15(3.05%) | 3(6.52%) |  |
| Albumin,g/L | 39.40(35.31-43.89) | 39.60(35.35-43.90) | 38.91(35.00-43.00) | 0.611 |
| ＜40 | 249(46.28%) | 224(45.53%) | 25(54.35%) | 0.709 |
| 40-55 | 220(40.89%) | 202(41.06%) | 18(39.13%) |  |
| ＞55 | 2(0.37%) | 2(0.41%) | 0(0%) |  |
| Globulin,g/L | 28.00(25.00-31.00) | 28.00(25.00-31.00) | 28.90(23.44-31.00) | 0.896 |
| ＜20 | 21(3.9%) | 18(3.66%) | 3(6.52%) | 0.681 |
| 20-40 | 444(82.53%) | 405(82.32%) | 39(84.78%) |  |
| ＞40 | 13(2.42%) | 12(2.44%) | 1(2.17%) |  |
| A/G | 1.41(1.25-1.62) | 1.41(1.26-1.60) | 1.38(1.17-1.70) | 0.991 |
| Prealbumin,mg/L | 194.70(122.50-251.00) | 193.98(122.50-254.08) | 201.00(116.70-242.83) | 0.739 |
| ＜150 | 79(14.68%) | 71(14.43%) | 8(17.39%) | 0.803 |
| 150-400 | 179(33.27%) | 159(32.32%) | 20(43.48%) |  |
| Ischemia modified albumin,U/ml | 75.67(69.66-80.70) | 75.67(69.33-80.75) | 76.55(72.45-.) | 0.719 |
| ＜85 | 19(3.53%) | 17(3.46%) | 2(4.35%) | 0.63 |
| ＞85 | 2(0.37%) | 2(0.41%) | 0(0%) |  |
| Total bilirubin,umol/L | 11.45(7.00-16.30) | 11.45(7.00-16.29) | 11.45(7.15-17.48) | 0.545 |
| ＜1.7 | 1(0.19%) | 1(0.2%) | 0(0%) | 0.709 |
| 1.7-20 | 402(74.72%) | 368(74.8%) | 34(73.91%) |  |
| ＞20 | 71(13.2%) | 63(12.8%) | 8(17.39%) |  |
| Direct bilirubin, umol/L | 3.40(2.10-5.30) | 3.40(2.10-5.30) | 3.18(2.05-5.65) | 0.896 |
| ＜1 | 25(4.65%) | 25(5.08%) | 0(0%) | 0.001 |
| 1-14 | 422(78.44%) | 384(78.05%) | 38(82.61%) |  |
| ＞14 | 6(1.12%) | 3(0.61%) | 3(6.52%) |  |
| Indirect bilirubin,umol/L | 7.40(4.54-11.10) | 7.40(4.49-11.00) | 7.40(4.90-11.86) | 0.502 |
| ＜15 | 402(74.72%) | 366(74.39%) | 36(78.26%) | 0.876 |
| ＞15 | 52(9.67%) | 47(9.55%) | 5(10.87%) |  |
| Low-density lipoprotein,mmol/L | 2.41(1.96-3.00) | 2.41(1.94-3.00) | 2.69(2.10-3.19) | 0.304 |
| ＜4.11 | 239(44.42%) | 216(43.9%) | 23(50%) | 0.514 |
| ＞4.11 | 4(0.74%) | 4(0.81%) | 0(0%) |  |
| High density lipoprotein,mmol/L | 1.03(0.84-1.25) | 1.03(0.86-1.25) | 0.94(0.83-1.26) | 0.682 |
| ＜0.9 | 75(13.94%) | 66(13.41%) | 9(19.57%) | 0.59 |
| 0.9-2.5 | 169(31.41%) | 155(31.5%) | 14(30.43%) |  |
| ＞2.5 | 2(0.37%) | 2(0.41%) | 0(0%) |  |
| Total cholesterol, mmol/L | 4.10(3.54-4.81) | 4.05(3.53-4.77) | 4.51(4.08-5.32) | 0.019 |
| ＜2.8 | 21(3.9%) | 20(4.07%) | 1(2.17%) | 0.794 |
| 2.8-6 | 299(55.58%) | 272(55.28%) | 27(58.7%) |  |
| ＞6 | 21(3.9%) | 19(3.86%) | 2(4.35%) |  |
| Creatine kinase,IU/L | 54(36.8-84.19) | 53.22(36.35-83.45) | 65(38.99-92.86) | 0.376 |
| ＜25 | 32(5.95%) | 28(5.69%) | 4(8.7%) | 0.309 |
| 25-190 | 303(56.32%) | 274(55.69%) | 29(63.04%) |  |
| ＞190 | 20(3.72%) | 16(3.25%) | 4(8.7%) |  |
| Amylase,U/L | 54.50(42.36-67.38) | 54.50(42.33-67.43) | 53.50(44.56-67.00) | 0.902 |
| ＜140 | 162(30.11%) | 144(29.27%) | 18(39.13%) |  |
| Lipase,U/L | 39.70(29.65-50.40) | 37.50(29.03-50.20) | 44.30(38.60-56.70) | 0.189 |
| ＜60 | 59(10.97%) | 53(10.77%) | 6(13.04%) | 0.625 |
| ＞60 | 6(1.12%) | 5(1.02%) | 1(2.17%) |  |
| Adenosine deaminase,IU/L | 11.70(9.00-14.00) | 11.30(9.00-14.00) | 13.10(8.11-14.13) | 0.541 |
| ＜4Iu/L | 1(0.19%) | 1(0.2%) | 0(0%) | 0.619 |
| 4-20Iu/L | 159(29.55%) | 137(27.85%) | 22(47.83%) |  |
| ＞20Iu/L | 5(0.93%) | 5(1.02%) | 0(0%) |  |
| Triglyceride, mmol/L | 1.38(0.95-2.05) | 1.35(0.91-2.01) | 1.85(1.40-2.64) | 0.002 |
| 0.24-1.86 | 219(40.71%) | 206(41.87%) | 13(28.26%) | 0.054 |
| ＞1.86 | 98(18.22%) | 86(17.48%) | 12(26.09%) |  |
| Triacylglycerol, mmol/L | 1.43(0.88-2.27) | 0.99(0.65-2.42) | 1.64(1.40-1.95) | 0.544 |
| ＜1.7 | 9(1.67%) | 7(1.42%) | 2(4.35%) | 0.771 |
| ＞1.7 | 7(1.3%) | 5(1.02%) | 2(4.35%) |  |
| Urea, mmol/L | 3.9(3.1-4.91) | 3.9(3.1-4.8) | 4.1(2.94-5.72) | 0.558 |
| ＜2.5 | 40(7.43%) | 37(7.52%) | 3(6.52%) | 0.682 |
| 2.5-8.2 | 360(66.91%) | 328(66.67%) | 32(69.57%) |  |
| ＞8.2 | 13(2.42%) | 11(2.24%) | 2(4.35%) |  |
| Uric acid,umol/L | 279.00(214.00-349.00) | 276.36(216.00-348.04) | 316.00(192.08-369.00) | 0.443 |
| ＜140 | 28(5.2%) | 24(4.88%) | 4(8.7%) | 0.138 |
| 140-440 | 387(71.93%) | 356(72.36%) | 31(67.39%) |  |
| ＞440 | 36(6.69%) | 30(6.1%) | 6(13.04%) |  |
| Creatinine, umol/L | 61.70(52.00-75.00) | 61.61(52.00-75.00) | 63.00(50.15-84.40) | 0.479 |
| ＜53 | 124(23.05%) | 113(22.97%) | 11(23.91%) | <0.0001 |
| 53-97 | 313(58.18%) | 289(58.74%) | 24(52.17%) |  |
| ＞97 | 16(2.97%) | 10(2.03%) | 6(13.04%) |  |
| Complement C1q,mg/L | 157.20(143.16-166.53) | 164.41(138.90-166.53) | 145.70(144.86-163.36) | 0.808 |
| ＜159 | 8(1.49%) | 5(1.02%) | 3(6.52%) | 0.248 |
| 159-233 | 8(1.49%) | 7(1.42%) | 1(2.17%) |  |
| α-L-fucosidase,U/L | 26.00(21.00-29.80) | 25.00(21.00-29.80) | 28.40(26.00-30.25) | 0.282 |
| ＜40 | 99(18.4%) | 87(17.68%) | 12(26.09%) | 0.459 |
| ＞40 | 4(0.74%) | 4(0.81%) | 0(0%) |  |
| 5‘nuclease, U/L | 3.00(2.00-3.00) | 3.00(2.50-4.00) | 2.00(2.00-2.75) | 0.066 |
| 5‘＜10 | 16(2.97%) | 12(2.44%) | 4(8.7%) | 0.567 |
| ＞10 | 1(0.19%) | 1(0.2%) | 0(0%) |  |
| Cystatin C, mg/L | 0.92(0.80-1.11) | 0.91(0.78-1.10) | 1.00(0.89-1.15) | 0.041 |
| ＜0.54 | 2(0.37%) | 2(0.41%) | 0(0%) | 0.119 |
| 0.54-1.5 | 164(30.48%) | 148(30.08%) | 16(34.78%) |  |
| ＞1.5 | 10(1.86%) | 7(1.42%) | 3(6.52%) |  |
| Urea/Creatinine | 61.63(5.55-90.47) | 65.75(34.82-90.36) | 55.73(0.48-104.15) | 0.575 |
| Glomerular filtration rate | 111.25(102.37-120.79) | 111.28(103.67-120.58) | 105.16(98.75-122.96) | 0.487 |
| ＞90 | 63(11.71%) | 57(11.59%) | 6(13.04%) | 0.58 |
| ＜90 | 6(1.12%) | 5(1.02%) | 1(2.17%) |  |
| Glucose,mmol/L | 5.55(4.85-6.79) | 5.55(4.85-6.76) | 6.11(4.85-7.45) | 0.386 |
| ＜3.57 | 4(0.74%) | 3(0.61%) | 1(2.17%) | 0.128 |
| 3.57-6.16 | 241(44.8%) | 225(45.73%) | 16(34.78%) |  |
| ＞6.16 | 138(25.65%) | 122(24.8%) | 16(34.78%) |  |
| kmmol/L | 3.90(3.59-4.20) | 3.9(3.59-4.20) | 3.94(3.62-4.12) | 0.838 |
| ＜3.5 | 72(13.38%) | 67(13.62%) | 5(10.87%) | 0.556 |
| 3.5-5.3 | 370(68.77%) | 334(67.89%) | 36(78.26%) |  |
| ＞5.3 | 6(1.12%) | 6(1.22%) | 0(0%) |  |
| Na,mmol/L | 139.00(137.00-141.00) | 139.00(137.00-141.00) | 139.60(138.00-141.50) | 0.041 |
| ＜137 | 99(18.4%) | 95(19.31%) | 4(8.7%) | 0.125 |
| 137-147 | 336(62.45%) | 300(60.98%) | 36(78.26%) |  |
| ＞147 | 8(1.49%) | 7(1.42%) | 1(2.17%) |  |
| Ca, mmol/L | 2.24(2.11-2.36) | 2.23(2.11-2.35) | 2.29(2.12-2.40) | 0.289 |
| ＜2.1 | 89(16.54%) | 81(16.46%) | 8(17.39%) | 0.722 |
| 2.1-2.8 | 324(60.22%) | 293(59.55%) | 31(67.39%) |  |
| ＞2.8 | 6(1.12%) | 6(1.22%) | 0(0%) |  |
| Cl,mmol/L | 102.80(100.00-105.00) | 102.90(100.00-105.00) | 101.70(98.28-103.55) | 0.063 |
| ＜99 | 62(11.52%) | 51(10.37%) | 11(23.91%) | 0.045 |
| 99-110 | 373(69.33%) | 343(69.72%) | 30(65.22%) |  |
| ＞110 | 3(0.56%) | 3(0.61%) | 0(0%) |  |
| C02, mmol/L | 25.30(24.00-27.00) | 25.50(24.00-27.00) | 25.00(23.65-27.47) | 0.703 |
| ＜18 | 4(0.74%) | 4(0.81%) | 0(0%) | 0.765 |
| 18-31 | 263(48.88%) | 235(47.76%) | 28(60.87%) |  |
| ＞31 | 12(2.23%) | 11(2.24%) | 1(2.17%) |  |
| P, mmol/L | 1.04(0.87-1.23) | 1.04(0.87-1.22) | 0.95(0.89-1.27) | 0.914 |
| ＜0.73 | 17(3.16%) | 16(3.25%) | 1(2.17%) | 0.734 |
| 0.73-1.55 | 173(32.16%) | 156(31.71%) | 17(36.96%) |  |
| ＞1.55 | 6(1.12%) | 5(1.02%) | 1(2.17%) |  |
| Total osmotic pressure, mOSM/l | 295.10(291.00-299.00) | 295.20(291.80-299.03) | 291.00(282.21-298.70) | 0.219 |
| ＜280 | 4(0.74%) | 3(0.61%) | 1(2.17%) | 0.351 |
| 280-320 | 79(14.68%) | 71(14.43%) | 8(17.39%) |  |
| Blood lactic acid, mmol/L | 2.90(2.90-2.90) | 2.90(2.90-2.90) | 0(0-0) |  |
| Anion gap,mmol/L | 10.50(8.85-12.20) | 10.20(8.50-12.00) | 13.00(9.40-16.30) | 0.011 |
| ＜10 | 45(8.36%) | 42(8.54%) | 3(6.52%) | 0.011 |
| 10-14 | 57(10.59%) | 52(10.57%) | 5(10.87%) |  |
| ＞14 | 7(1.3%) | 4(0.81%) | 3(6.52%) |  |
| White blood cell count, 10^9/L | 5.86(4.43-7.50) | 5.84(4.5-7.49) | 5.98(4.3-7.69) | 0.78 |
| ＜3.5 | 44(8.18%) | 39(7.93%) | 5(10.87%) | 0.725 |
| 3.5-9.5 | 386(71.75%) | 354(71.95%) | 32(69.57%) |  |
| ＞9.5 | 48(8.92%) | 43(8.74%) | 5(10.87%) |  |
| Neutrophil count,10^9/L | 3.64(2.71-5.27) | 3.66(2.71-5.26) | 3.60(2.28-5.96) | 0.994 |
| ＜1.8 | 35(6.51%) | 31(6.3%) | 4(8.7%) | 0.706 |
| 1.8-6.3 | 365(67.84%) | 335(68.09%) | 30(65.22%) |  |
| ＞6.3 | 77(14.31%) | 69(14.02%) | 8(17.39%) |  |
| Neutrophil percentage, % | 65.4(56.7-74.6) | 65.4(56.73-73.98) | 64.8(53.7-78.8) | 0.641 |
| ＜40 | 16(2.97%) | 16(3.25%) | 0(0%) | 0.193 |
| 40-75 | 349(64.87%) | 320(65.04%) | 29(63.04%) |  |
| ＞75 | 114(21.19%) | 100(20.33%) | 14(30.43%) |  |
| Lymphocyte count, 10^9/L | 1.39(0.96-1.80) | 1.40(0.99-1.80) | 1.27(0.80-1.65) | 0.161 |
| ＜1.1 | 147(27.32%) | 130(26.42%) | 17(36.96%) | 0.198 |
| 1.1-3.2 | 322(59.85%) | 296(60.16%) | 26(56.52%) |  |
| ＞3.2 | 17(3.16%) | 17(3.46%) | 0(0%) |  |
| Lymphocyte percentage, % | 24.40(17.10-32.90) | 24.55(17.65-33.00) | 23.70(14.90-32.50) | 0.365 |
| ＜20 | 163(30.3%) | 145(29.47%) | 18(39.13%) | 0.324 |
| 20-50 | 314(58.36%) | 289(58.74%) | 25(54.35%) |  |
| ＞50 | 10(1.86%) | 10(2.03%) | 0(0%) |  |
| Monocyte count, 10^9/L | 0.43(0.33-0.56) | 0.43(0.33-0.57) | 0.43(0.32-0.52) | 0.611 |
| ＜1.1 | 402(74.72%) | 361(73.37%) | 41(89.13%) | 0.601 |
| 1.1-3.2 | 5(0.93%) | 5(1.02%) | 0(0%) |  |
| ＞3.2 | 4(0.74%) | 4(0.81%) | 0(0%) |  |
| Monocyte percentage% | 7.5(5.9-9.28) | 7.5(5.9-9.4) | 7.3(5.45-9.1) | 0.551 |
| ＜3 | 408(75.84%) | 367(74.59%) | 41(89.13%) | 0.677 |
| 3-10 | 6(1.12%) | 6(1.22%) | 0(0%) |  |
| ＞10 | 1(0.19%) | 1(0.2%) | 0(0%) |  |
| Eosinophil count, 10^9/L | 0.04(0.01-0.09) | 0.04(0.01-0.09) | 0.03(0-0.09) | 0.435 |
| ＜0.02 | 143(26.58%) | 127(25.81%) | 16(34.78%) | 0.48 |
| 0.02-0.52 | 264(49.07%) | 240(48.78%) | 24(52.17%) |  |
| ＞0.52 | 4(0.74%) | 3(0.61%) | 1(2.17%) |  |
| Eosinophil percentage, % | 0.60(0.10-1.60) | 0.60(0.10-1.60) | 0.30(0.05-1.70) | 0.382 |
| ＜0.4 | 164(30.48%) | 143(29.07%) | 21(45.65%) | 0.25 |
| 0.4-8 | 244(45.35%) | 224(45.53%) | 20(43.48%) |  |
| ＞8 | 4(0.74%) | 4(0.81%) | 0(0%) |  |
| Basophil count, 10^9/L | 0.01(0.01-0.02) | 0.01(0.01-0.02) | 0.01(0-0.03) | 0.215 |
| Basophil percentage, 10^9/L | 0.20(0.10-0.40) | 0.20(0.10-0.30) | 0.20(0.05-0.40) | 0.902 |
| ＜1 | 403(74.91%) | 362(73.58%) | 41(89.13%) | 0.452 |
| ＞1 | 5(0.93%) | 5(1.02%) | 0(0%) |  |
| Red blood cell count,10^12/L | 4.46(4.08-4.86) | 4.46(4.08-4.85) | 4.61(4.22-4.9) | 0.411 |
| ＜3.8 | 62(11.52%) | 59(11.99%) | 3(6.52%) | 0.462 |
| 3.8-5.1 | 353(65.61%) | 319(64.84%) | 34(73.91%) |  |
| ＞5.1 | 73(13.57%) | 67(13.62%) | 6(13.04%) |  |
| Haemoglobin, g/L | 134.00(122.25-148.00) | 134.00(121.00-148.00) | 138.50(125.50-147.50) | 0.369 |
| ＜115 | 76(14.13%) | 72(14.63%) | 4(8.7%) | 0.399 |
| 115-150 | 301(55.95%) | 271(55.08%) | 30(65.22%) |  |
| ＞150 | 103(19.14%) | 95(19.31%) | 8(17.39%) |  |
| Hematocrit, % | 39.50(34.80-43.80) | 39.35(34.83-43.80) | 40.40(33.05-43.75) | 0.91 |
| ＜35 | 121(22.49%) | 109(22.15%) | 12(26.09%) | 0.718 |
| 35-45 | 259(48.14%) | 236(47.97%) | 23(50%) |  |
| ＞45 | 89(16.54%) | 83(16.87%) | 6(13.04%) |  |
| Average volume of red blood cells,fl | 90.00(86.90-93.20) | 90.05(86.90-93.20) | 89.70(87.50-94.30) | 0.933 |
| Mean hemoglobin,pg | 30.40(29.20-31.40) | 30.30(29.18-31.40) | 30.60(29.20-31.40) | 0.677 |
| ＜27 | 45(8.36%) | 44(8.94%) | 1(2.17%) | 0.231 |
| 27-34 | 424(78.81%) | 383(77.85%) | 41(89.13%) |  |
| ＞34 | 16(2.97%) | 15(3.05%) | 1(2.17%) |  |
| Mean hemoglobin concentration,g/L | 335.00(326.00-344.00) | 335.00(326.00-344.00) | 334.00(327.00-343.00) | 0.679 |
| ＜316 | 45(8.36%) | 44(8.94%) | 1(2.17%) | 0.116 |
| 316-354 | 417(77.51%) | 379(77.03%) | 38(82.61%) |  |
| ＞354 | 24(4.46%) | 20(4.07%) | 4(8.7%) |  |
| Red blood cell distribution width SD,fL | 41.00(38.43-44.00) | 41.00(38.50-44.30) | 40.90(38.20-43.50) | 0.282 |
| ＜39 | 119(22.12%) | 106(21.54%) | 13(28.26%) | 0.469 |
| 39-46 | 218(40.52%) | 198(40.24%) | 20(43.48%) |  |
| ＞46 | 71(13.2%) | 67(13.62%) | 4(8.7%) |  |
| Red blood cell distribution width CV, % | 12.50(12.00-13.30) | 12.50(12.00-13.30) | 12.40(11.98-13.10) | 0.471 |
| ＜11.5 | 27(5.02%) | 26(5.28%) | 1(2.17%) | 0.597 |
| 11.5-14.5 | 389(72.3%) | 352(71.54%) | 37(80.43%) |  |
| ＞14.5 | 45(8.36%) | 41(8.33%) | 4(8.7%) |  |
| Platelet count,10^9/L | 201.00(152.50-258.75) | 200.00(151.00-258.25) | 213.00(158.50-270.25) | 0.587 |
| 125-350 | 454(84.39%) | 415(84.35%) | 39(84.78%) | 0.605 |
| ＞350 | 26(4.83%) | 23(4.67%) | 3(6.52%) |  |
| Platelet hematocrit, % | 0.21(0.17-0.26) | 0.21(0.17-0.26) | 0.21(0.15-0.26) | 0.839 |
| ＜0.17 | 88(16.36%) | 78(15.85%) | 10(21.74%) | 0.836 |
| 0.17-0.39 | 261(48.51%) | 237(48.17%) | 24(52.17%) |  |
| ＞0.39 | 11(2.04%) | 10(2.03%) | 1(2.17%) |  |
| Mean platelet volume, fl | 10.10(9.40-11.1) | 10.10(9.40-11.18) | 10.20(9.55-10.95) | 0.859 |
| ＜9 | 73(13.57%) | 69(14.02%) | 4(8.7%) | 0.204 |
| 9-13 | 351(65.24%) | 315(64.02%) | 36(78.26%) |  |
| Platelet distribution width | 15.50(11.60-16.40) | 15.50(11.50-16.40) | 14.80(11.85-16.10) | 0.405 |
| <9 | 5(0.93%) | 4(0.81%) | 1(2.17%) | 0.088 |
| 9~17 | 369(68.59%) | 331(67.28%) | 38(82.61%) |  |
| >17 | 56(10.41%) | 55(11.18%) | 1(2.17%) |  |
| Large platelet ratio, % | 27.65(22.60-34.38) | 27.70(22.50-35.15) | 26.50(23.55-30.90) | 0.576 |
| <13 | 2(0.37%) | 2(0.41%) | 0(0%) | 0.321 |
| 13~43 | 269(50%) | 241(48.98%) | 28(60.87%) |  |
| >43 | 35(6.51%) | 34(6.91%) | 1(2.17%) |  |
| Naive granulocyte percentage, % | 0.10(0.03-0.38) | 0.10(0.03-0.35) | 0.03(0.03-0.50) | 0.844 |
| ＜4 | 39(7.25%) | 32(6.5%) | 7(15.22%) | 0.641 |
| ＞4 | 1(0.19%) | 1(0.2%) | 0(0%) |  |
| Activated partial thromboplastin time, s | 30.35(26.20-34.75) | 30.55(26.68-34.93) | 29.40(25.75-34.60) | 0.321 |
| ＜20 | 6(1.12%) | 4(0.81%) | 2(4.35%) | 0.083 |
| 20~40 | 224(41.64%) | 203(41.26%) | 21(45.65%) |  |
| ＞40 | 26(4.83%) | 25(5.08%) | 1(2.17%) |  |
| Prothrombin time, s | 16.50(15.00-18.00) | 16.40(15.00-18.00) | 16.80(14.60-19.15) | 0.484 |
| 8~16 | 114(21.19%) | 105(21.34%) | 9(19.57%) | 0.477 |
| ＞16 | 153(28.44%) | 137(27.85%) | 16(34.78%) |  |
| Fibrinogen, g/L | 3.13(2.46-3.80) | 3.15(2.39-3.84) | 2.89(2.58-3.52) | 0.781 |
| ＜1.7 | 9(1.67%) | 9(1.83%) | 0(0%) | 0.508 |
| 1.7~4 | 203(37.73%) | 182(36.99%) | 21(45.65%) |  |
| ＞4 | 53(9.85%) | 49(9.96%) | 4(8.7%) |  |
| Prothrombin time, s | 12.30(11.50-13.30) | 12.35(11.60-13.30) | 11.90(11.10-13.25) | 0.26 |
| ＜9.5 | 2(0.37%) | 2(0.41%) | 0(0%) | 0.899 |
| 9.5~14.5 | 242(44.98%) | 219(44.51%) | 23(50%) |  |
| ＞14.5 | 22(4.09%) | 20(4.07%) | 2(4.35%) |  |
| D-dimer,ug/mL | 0.40(0.23-0.80) | 0.40(0.24-0.80) | 0.40(0.21-0.85) | 0.996 |
| D＜200 | 184(34.2%) | 164(33.33%) | 20(43.48%) | 0.727 |
| D＞200 | 1(0.19%) | 1(0.2%) | 0(0%) |  |
| Erythrocyte sedimentation rate, MM/h | 22.50(10.00-36.00) | 22.00(10.00-35.00) | 32.50(8.28-48.75) | 0.334 |
| 0~20 | 59(10.97%) | 54(10.98%) | 5(10.87%) | 0.786 |
| ＞20 | 71(13.2%) | 64(13.01%) | 7(15.22%) |  |
| C-reactive protein,mg/L | 7.87(2.27-24.75) | 7.70(2.26-25.98) | 9.30(4.51-20.27) | 0.882 |
| ＜10 | 108(20.07%) | 98(19.92%) | 10(21.74%) | 0.988 |
| ＞10 | 87(16.17%) | 79(16.06%) | 8(17.39%) |  |
| Hypersensitive C-reactive protein, mg/L | 2.49(1.38-6.18) | 2.49(1.50-6.18) | 2.87(0.88-7.17) | 0.526 |
| ＜0.5 | 8(1.49%) | 6(1.22%) | 2(4.35%) | 0.215 |
| ＞0.5 | 96(17.84%) | 86(17.48%) | 10(21.74%) |  |
| Procalcitonin, ng/ml | 0.09(0.05-0.14) | 0.10(0.05-0.14) | 0.08(0.05-0.10) | 0.718 |
| ＜0.5 | 109(20.26%) | 95(19.31%) | 14(30.43%) | 0.482 |
| ＞0.5 | 4(0.74%) | 3(0.61%) | 1(2.17%) |  |
| Hypersensitive troponinpg/ml | 0.90(0.03-9.60) | 2.95(0.03-9.60) | 0.06(0.02-.) | 0.855 |
| 0~14 | 21(3.9%) | 19(3.86%) | 2(4.35%) | 0.104 |
| ＞14 | 2(0.37%) | 1(0.2%) | 1(2.17%) |  |
| Myoglobin, ng/ml | 25.00(17.63-49.25) | 25.00(18.00-50.70) | 18.70(15.05-39.80) | 0.396 |
| ＜25 | 37(6.88%) | 34(6.91%) | 3(6.52%) | 0.871 |
| 25~38 | 19(3.53%) | 18(3.66%) | 1(2.17%) |  |
| ＞38 | 20(3.72%) | 19(3.86%) | 1(2.17%) |  |
| Body temperature during blood collection | 36.60(36.30-36.80) | 36.60(36.30-36.80) | 36.50(35.50-.) | 0.6 |
| Blood oxygen concentration | 29.00(21.00-29.00) | 29.00(21.00-29.00) | 29.00(29.00-29.00) | 0.453 |
| PH | 7.41(7.38-7.44) | 7.41(7.38-7.44) | 7.42(7.37-7.45) | 0.892 |
| ＜7.35 | 1(0.19%) | 1(0.2%) | 0(0%) | 0.942 |
| 7.35~7.45 | 64(11.9%) | 61(12.4%) | 3(6.52%) |  |
| ＞7.45 | 16(2.97%) | 15(3.05%) | 1(2.17%) |  |
| PH (T correction) | 7.40(7.38-7.43) | 7.40(7.38-7.42) | 7.45(7.45-7.45) | 0.163 |
| CO2 partial pressure | 39.60(34.00-42.63) | 39.60(34.15-42.55) | 38.00(31.45-43.75) | 0.818 |
| ＜35 | 20(3.72%) | 19(3.86%) | 1(2.17%) | 0.757 |
| 35~45 | 59(10.97%) | 55(11.18%) | 4(8.7%) |  |
| ＞45 | 7(1.3%) | 7(1.42%) | 0(0%) |  |
| Carbon dioxide partial pressure (T correction), mmHg | 41.00(37.60-44.10) | 41.00(37.85-43.85) | 37.05(29.80-.) | 0.708 |
| ＜75 | 43(7.99%) | 41(8.33%) | 2(4.35%) |  |
| Oxygen partial pressure | 91.25(74.00-115.75) | 91.50(74.00-117.50) | 78.20(65.95-110.00) | 0.455 |
| ＜80 | 33(6.13%) | 30(6.1%) | 3(6.52%) | 0.58 |
| 80~100 | 22(4.09%) | 21(4.27%) | 1(2.17%) |  |
| ＞100 | 31(5.76%) | 30(6.1%) | 1(2.17%) |  |
| Oxygen partial pressure (T correction), mmHg | 109.80(85.00-163.00) | 109.80(86.40-163.50) | 101.90(80.80-.) | 0.686 |
| <75 | 6(1.12%) | 6(1.22%) | 0(0%) | 0.609 |
| 75~100 | 10(1.86%) | 9(1.83%) | 1(2.17%) |  |
| ＞100 | 27(5.02%) | 26(5.28%) | 1(2.17%) |  |
| Whole blood remaining alkali, mmol/L | 0.20(-1.00-1.65) | 0.20(-1.00-1.78) | 0.20(-3.65-1.05) | 0.449 |
| ＜-3 | 3(0.56%) | 2(0.41%) | 1(2.17%) | 0.102 |
| -3~3 | 75(13.94%) | 71(14.43%) | 4(8.7%) |  |
| ＞3 | 7(1.3%) | 7(1.42%) | 0(0%) |  |
| Plasma remaining alkali, mmol/L | 0.55(-0.33-1.58) | 0.60(-0.25-1.65) | -1.2(-1.2--1.2) | 0.144 |
| Arterial oxygen content, ml/dl | 16.80(14.65-19.20) | 16.70(14.68-18.98) | 19.20(13.00-.) | 0.699 |
| ＜6.7 | 1(0.19%) | 1(0.2%) | 0(0%) | 0.928 |
| 6.7~10.3 | 1(0.19%) | 1(0.2%) | 0(0%) |  |
| ＞10.3 | 43(7.99%) | 40(8.13%) | 3(6.52%) |  |
| Oxygen saturation, % | 97.10(96.00-99.00) | 97.80(96.00-99.00) | 96.60(95.30-97.00) | 0.288 |
| ＜92.5 | 4(0.74%) | 4(0.81%) | 0(0%) | 0.305 |
| 92.5~98.5 | 48(8.92%) | 44(8.94%) | 4(8.7%) |  |
| ＞95.8 | 23(4.28%) | 23(4.67%) | 0(0%) |  |
| Oxygen partial pressure / oxygen concentration ratio, mmHg | 452.00(305.50-570.50) | 458.50(300.75-571.75) | 425.00(425.00-425.00) | 0.708 |
| Alveolar-arterial oxygen partial pressure difference, kpa | 30.55(12.53-57.70) | 30.50(11.75-57.70) | 33.80(12.10-.) | 0.539 |
| Arterial / alveolar oxygen partial pressure | 81.00(61.55-104.40) | 84.00(60.58-104.60) | 78.50(78.50-78.50) | 0.779 |
| Total hemoglobin, g/L | 19.10(14.15-113.50) | 40.10(13.63-116.00) | 15.50(14.55-72.18) | 0.397 |
| ＜120 | 52(9.67%) | 48(9.76%) | 4(8.7%) | 0.564 |
| 120~160 | 10(1.86%) | 10(2.03%) | 0(0%) |  |
| ＞160 | 4(0.74%) | 4(0.81%) | 0(0%) |  |
| **Imaging features** | | | | |
| Normal | 45(8.36%) | 43(8.74%) | 2(4.35%) | 0.500 |
| One side ground-glass opacity | 94(17.47%) | 88(17.89%) | 6(13.04%) |  |
| Both sides ground-glass opacity | 350(65.06%) | 316(64.23%) | 34(73.91%) |  |
| Consolidation | 11(2.04%) | 11(2.24%) | 0(0%) |  |
| Others | 38(7.06%) | 34(6.91%) | 4(8.7%) |  |
| Data are median (IQR), n (%), or n/N (%). p values were calculated by Mann-Whitney U test, χ² test, or Fisher’s exact test, as appropriate. | | | | |

| **Supplementary Table 4: Risk factors associated with COVID-19 patients with diabetes in generalized linear model** | | | |
| --- | --- | --- | --- |
| **Parameter** | **Estimate** | **Standard error** | ***P* value** |
| intercept | 0.3528 | 0.4417 | 0.43 |
| Glucose,mmol/L | 0.4033 | 0.1845 | 0.03 |
| Total cholesterol 2.8-6, mmol/L | 0.0154 | 0.0667 | 0.82 |
| Age | 0.0035 | 0.0037 | 0.34 |
| Prothrombin time 9.5~14.5, s | -0.0593 | 0.0248 | 0.02 |
| Sex | 0.1323 | 0.1004 | 0.19 |
